# Psychological distress of adult patients consulting a center for rare and undiagnosed diseases: a cross-sectional study

**DOI:** 10.1186/s13023-023-02669-7

**Published:** 2023-04-14

**Authors:** Meike Mund, Natalie Uhlenbusch, Franziska Rillig, Christina Weiler-Normann, Theresia Herget, Christian Kubisch, Bernd Löwe, Christoph Schramm

**Affiliations:** 1 Martin Zeitz Center for Rare Diseases, University Medical Center Hamburg-Eppendorf, Hamburg, Germany; 2 Department of Psychosomatic Medicine and Psychotherapy, University Medical Center Hamburg-Eppendorf, Martinistraße 52, Hamburg, 20246 Germany; 3 1st Department of Medicine, University Medical Center Hamburg-Eppendorf, Hamburg, Germany; 4 Institute of Human Genetics, University Medical Center Hamburg-Eppendorf, Hamburg, Germany

**Keywords:** Rare diseases, Center for rare diseases, Mental health, Persistent somatic symptoms, Psychological distress

## Abstract

**Background:**

Centers for rare diseases serve as contact points for patients with complex, often undiagnosed complaints and persistent somatic symptoms of heterogeneous origin. Little is known about psychological distress of patients consulting these centers.

**Objectives:**

To better understand psychological distress of adult patients presenting at a center for rare diseases by determining the proportion of patients screening positive for depressive, anxiety, and somatic symptom disorders (SSD) and to identify factors associated with increased psychopathology.

**Methods:**

Cross-sectional data from the routine care registry of the Martin Zeitz Center for Rare Diseases (MZCSE) at the University Medical Center Hamburg-Eppendorf in Germany was retrieved and analyzed. We included all adult patients presenting between October 01,2020 and September 30,2021, who gave written informed consent.

**Measures:**

Sociodemographic variables, medical history and healthcare utilization, as well as validated measures to screen for a depressive disorder (PHQ-8), an anxiety disorder (GAD-7), and SSD (PHQ-15, SSD-12).

**Results:**

*N* = 167 patients were included (age 44.5 ± 14.3 years, 64.7% female). A total of 40.7% of the patients screened positive for a depressive disorder (PHQ-8 ≥ 10), 27.5% for an anxiety disorder (GAD-7 ≥ 10) and 45.0% screened positive for SSD (PHQ-15 ≥ 9 & SSD-12 ≥ 23). Factors associated with increased psychopathology included the number of symptoms, the number of different specialties consulted before and past psychotherapy.

**Conclusions:**

Patients presenting at centers for rare diseases are likely to experience high rates of psychological distress. Systematically screening patients with rare and undiagnosed diseases for mental disorders can help to detect those at risk at an early stage and initiate adequate psychological care.

**Supplementary Information:**

The online version contains supplementary material available at 10.1186/s13023-023-02669-7.

## Introduction

Centers for rare and undiagnosed diseases serve as contact points for patients with an unclear diagnosis. These centers have been established in several countries over the past decade as part of many efforts to improve care for patients with rare diseases [1]. Patients mostly present with complex and multiple persistent somatic symptoms of unknown aetology. Three recent studies [2–4] gave first insights into this patient population. In a monocentric study from Germany, the majority of the patients presenting to the center suffered from several unspecific somatic symptoms, mostly general weakness and fatigue as well as pain. The three most frequent diagnoses were soft tissue disorders, somatoform disorders, and polyneuropathies [2]. In an undiagnosed disease program in the US, Waserstein and colleagues [3] found at least one psychiatric symptom in 72% of the patients, with 24.3% having a pre-existing psychiatric diagnosis. Patients with a psychiatric symptom had significantly lower quality of life enjoyment and satisfaction than patients without a psychiatric symptom [3]. In a multicenter study [4], the results of the diagnostic process across ten centers for rare diseases are described: Of *N* = 2033 adult patients without a diagnosis, *n* = 521 (26%) received one after presenting at the center. Of these, 60% were classified as rare diseases, 23% as common diseases and 17% as psychosomatic diseases (e.g. somatization disorder). For the remaining patients, the origin of their mostly persistent somatic symptoms remained unclear. Regardless of the diagnostic outcome, the often long diagnostic process can be very challenging for the patients [4]. However, little is known about psychological distress of patients presenting at centers for rare diseases.

In diagnosed rare diseases, the challenges patients face often lead to high psychological distress [5, 6]. A rare disease is defined as affecting less than 1 in 2000 people and it is estimated that 6000–8000 rare diseases exist [7]. Despite the low prevalence of rare diseases, the number of individuals affected by any rare disease is high with around 300 million worldwide, making rare diseases a major public health issue [8]. Patients experience substantial delay in diagnosis and access to adequate care is frequently limited [9]. For affected individuals, rare diseases are often associated with diverse functional, social, and psychological consequences [10, 11]. Uhlenbusch and colleagues [5] conducted a systematic review and meta-analysis to estimate the frequency of affective and anxiety disorders in patients with any rare disease and considered 39 studies including *N* = 5951 patients with 24 different rare diseases. Among the included conditions, the authors found high prevalence rates for both depressive and anxiety disorders, with pooled prevalence estimates of 13.1% for current and 39.3% for lifetime major depressive disorder and 39.6% for current and 44.2% for lifetime anxiety disorders [5]. In a cross-sectional study, Uhlenbusch and colleagues [6] examined mental health of *N* = 300 patients with 79 different rare diseases and found moderately or severely elevated depression and anxiety levels in 42% and 23% of the patients, respectively. Comorbid mental diseases in patients with chronic conditions can worsen the course of the disease [12] and contribute to reduced quality of life [13, 14].

Independent of whether a diagnosed rare disease is causal for patients’ somatic complaints, persistent somatic symptoms can lead to high psychological distress [15]. Persistent somatic symptoms is an umbrella term for somatic complaints that are present on most days over a period of several months and subjectively distressing, regardless of their aetiology. The term comprises different bodily complaints like dizziness, palpitations, diarrhoea, pain, fatigue, and many more [16]. Anxiety, depression, and somatization frequently appear together and the overlap contributes exceedingly to functional impairment [17]. Persistent somatic symptoms, independent of their origin, are associated with impairment [18], functional limitations, and reduced physical and mental quality of life [19]. Anxiety disorders, depressive disorders and somatic symptom disorders (SSD) are the most prevalent mental disorders in patients with persistent somatic symptoms [20]. The prevalence of SSD, as the successor of the diagnostic concept of somatoform disorders, has only been investigated in few studies, while for many clinical populations the frequency is still unclear [20]. For patients consulting a center for rare diseases, neither the prevalences of SSD nor of anxiety and depressive disorders has been described yet.

The diagnostic process can be a considerable burden for patients with rare diseases. Among the experiences that patients describe as burdening is the feeling of not being taken seriously or being labelled as psychosomatic or hypochondriacal [21]. This misconception is rooted in a biomedical understanding of disease. Following a biomedical approach, patients are diagnosed as *either* somatically *or* mentally ill. The absence of a somatic explanation for persistent somatic symptoms therefore inevitably results in the label of being mentally ill, ignoring biopsychosocial interrelationships. The diagnostic concept of SSD allows to describe mental distress due to somatic symptoms, regardless of whether these symptoms are caused by a somatic illness or not. It can therefore help to overcome mind-body dualism and reduce stigmatization of patients [20].

Taken together, the evidence illustrated above demonstrates the complexity and heterogeneity of patients consulting centers for rare and undiagnosed diseases and indicates a high likelihood of psychological distress. Regardless of the diagnostic outcome, patients are likely to experience psychological burden at presentation. Detecting psychological distress and mental diseases at an early stage and foster rapid initiation of appropriate treatments can be crucial for patients’ overall health and well-being. Better understanding psychological characteristics of patients consulting a center for rare diseases can therefore improve comprehensive care. With the current study, we aimed to examine psychological distress in adult patients consulting the Martin Zeitz Center for Rare Diseases (MZCSE) at the University Medical Center Hamburg-Eppendorf in Germany. More specifically, we sought to determine the proportion of patients screening positive for a depressive disorder, an anxiety disorder, and SSD and identify factors that are associated with increased psychopathology.

## Methods

### Study design

This cross-sectional study aimed to investigate characteristics of consecutive patients presenting in routine care at the MZCSE between October 01, 2020 and September 30, 2021. This study follows the STrengthening the Reporting of OBservational studies in Epidemiology (STROBE) criteria [22].

### Routine procedure at the MZCSE

Referring physicians, usually primary care physicians suspecting a rare disease in their patients, hand in a medical epicrisis with the main symptoms and, if applicable, a suspected diagnosis. According to the epicrisis, physicians of the MZCSE judge the possibility of a rare disease. In case of consideration, the patients are asked to hand in their full medical history and complete a paper-pencil-survey. The survey assesses demographics, socioeconomic status, medical history, health care utilization, diagnostic examinations, medication and psychopathology of the patients and is entered into the MZCSE-registry by trained research assistants. It is cross-checked for correctness and participants’ responses deviating from the permissible format of the questions are treated as missing values. The data is entered pseudonymized and patients give written informed consent. This procedure did not change during the COVID-19 pandemic.

### Study population

Every patient who was at least 18 years old and returned the survey to the MZCSE during the study period with informed consent, was eligible for the study.

### Data collection

We retrieved data from the MZCSE-registry from all patients who returned the survey from October 01, 2020 to September 30, 2021. The independent ethics committee of the Hamburg Medical Chamber issued a positive ethics vote for the MZCSE-registry on March 25, 2019. For the retrieval of data analyzed in this study, we received a further positive ethics vote (PV6022).

### Variables

Considered variables were demographics (sex, age), socioeconomic status (highest education, employment status), medical history (comorbidities, symptoms, time since symptom onset, times and days in stationary care, past psychotherapeutic treatment), healthcare utilization (time since first physician contact with regard to symptoms, number of consulted disciplines), and psychopathology (screening for depressive and anxiety disorders and SSD). Demographics and socioeconomic status were determined with categorical questions. Symptoms, comorbidities, and consulted disciplines were collected with multi-response sets. Time since symptom onset and time since first physician contact with regard to symptoms were assessed with dates. Validated questionnaires were used to screen for a depressive disorder, an anxiety disorder, and SSD:

#### Depression screening

Symptoms of a depressive disorder were measured using the German version of the depression module of the Patient Health Questionnaire-8 (PHQ-8) [23], an 8-item screening instrument determining severity levels of depressive symptoms from 0 (not bothered at all) to 3 (bothered almost every day). As a single cut-off value indicating a depressive disorder, the authors recommend a sum-score of **≥** 10 [24]. The instrument demonstrates satisfactory internal consistency and validity in several populations [25–27].

#### Anxiety screening

Symptoms of an anxiety disorder were assessed using the German version of the Generalized Anxiety Disorder 7-item scale (GAD-7) [28, 29]. The GAD-7 can be used in diagnostic procedures to detect generalized anxiety disorder as well as for screening for any other anxiety disorder [30]. A sum-score value of ≥ 10 indicates an anxiety disorder [28]. The GAD-7 demonstrates good internal consistency (Cronbach’s α = 0.89) [29], good test-retest reliability (intraclass correlations = 0.83) as well as criterion, construct, factorial, and procedural validity [28].

#### SSD screening

To screen for SSD, two scales were combined, assessing somatic symptom severity on the one hand and patients’ perceptions of their symptom-related thoughts, feelings, and behaviors on the other. Somatic symptom severity was assessed using the German version of the PHQ-15 [31, 32]. The instrument is composed of 15 items measuring somatic symptoms, each symptom scored from 0 (“not bothered at all”) to 2 (“bothered a lot”). It is a valid and reliable instrument to assess somatic symptom burden and screen for somatoform disorders [33]. Scores of ≥ 5, ≥10, ≥ 15 refer to mild, moderate, and severe somatic symptom severity, respectively. As a single cut-off a score of ≥ 10 reflects medium somatic symptom severity [31, 34].

Patients’ perceptions of their symptom-related thoughts, feelings, and behaviors were measured using the Somatic Symptom Disorder-B Criteria Scale (SSD-12) [35]. The scale was developed after the new diagnosis of SSD was introduced in the DSM-5 in order to assess the newly added B-criterion. SSD replaced DSM IV’s somatization disorders and the B-criterion describes psychological burden through somatic symptoms on a cognitive, emotional, and behavioral level. Besides the formulaton of the positive psychological B-criterion, the main difference is that the exclusion of an underlying cause for the somatic symptoms is no longer necessary, allowing to diagnose patients regardless of the origin of their somatic symptoms. The 12-item instrument has an excellent internal consistency (Cronbach’s α = 0.95) [35]. Validation of the SSD-12 in primary care showed that it is reliable and valid in measuring psychological characteristics, which are related to the experience of somatic symptoms [36]. Cut-off values of the SSD-12 depend on gender and age [37]. The PHQ-15 and SSD-12 combined are used to screen for SSD. Here, a score of 9 and higher in the PHQ-15 combined with a score of 23 and higher in the SSD-12 indicates SSD (sensitivity = 69%, specificity = 70%) [38].

### Data analysis

We calculated descriptive measures for metric variables (mean, standard deviation, range) and for categorical variables (frequencies, valid percentages). Further, we determined percentages of valid cases of patients screening positive for a depressive disorder (PHQ-8), an anxiety disorder (GAD-7), and SSD (PHQ-15, SSD-12). Missing data in single items of the PHQ-8, GAD-7, and PHQ-15 were imputated with mean values, if more than 80% of the answers were given, which is recommended for the Patient Health Questionnaire [33]. Data of the SSD-12 were imputated with mean values, if 9 or more values were given [35]. To exploratorily determine aspects that are associated with screening positive for a depressive disorder, anxiety disorder or SSD (treated as binary variables with 1 = being above the cut-off and 0 = being below the cut-off), we calucated Chi-Square/Fisher’s exact tests for categorical variables and *t*-tests for continuous variables. In case assumptions for the use of parametric tests were not met, we used the Mann-Whitney U test as non-parametric alternative. If a subgroup of a categorical variable had less than 10 cases, it was excluded from this analysis. Due to the exploratory character of this analysis, we refrained from alpha error correction. All tests were perfomed two-sided and *p* < .05 was considered statistically significant. Data was analyzed using IBM SPSS 27 [39]. BioVenn was used to determine overlaps of patients in groups and create an area-proportional Venn diagram [40].

## Results

### Case numbers

**Fig. 1:**
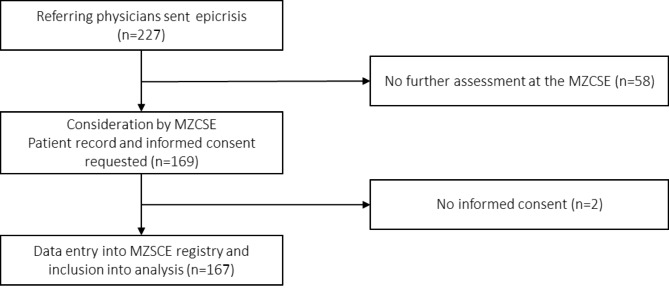
Case numbers in the different stages of the routine patient flow at the MZCSE between 01/10/2020 and 30/09/2021

A total of 169 consecutive patients presenting at the MZCSE were considered. Two patients did not give informed consent to the MZCSE-registry. Data of the remaining *N* = 167 patients (98.8%) were analyzed. Figure 1 shows the patient flow.

### Sociodemographic and clinical characteristics

The mean age of the patients was *M* = 44.5 years (*SD* = 14.3, range 18–75) and 64.7% were female. A slight majority of the patients (53.3%) completed an apprenticeship and 36.4% attended university. At the time of presentation, 57.4% of the patients were employed, 6.1% still in education, 8.8% were on retirement pension and 12.2% on disability pension. About a third of the patients (34.0%) were on sick-leave at time of survey completion with a mean duration of 55.1 weeks (*SD* = 71.7; range 0-374). A detailed overview of the sociodemographic characteristics is displayed in Table 1.

**Table 1:** Sociodemographic characteristics

Demographics	*M* (*SD*) / *n* (%)
**Age**	
mean (SD)	44.5 (14.3)
median (range)	44.0 (18–75)
**Age categories**	
19 and younger	2 (1.2)
20–29	26 (15.6)
30–39	40 (24.0)
40–49	31 (18.6)
50–59	40 (24.0)
60–69	21 (12.6)
70 and older	7 (4.2)
**Gender**	
female	108 (65.1)
male	58 (34.9)
missing	1
**Highest school graduation**	
still going to school	1 (0.6)
graduation after 9th grade	22 (13.6)
graduation after 10th grade	41 (25.3)
technical baccalaureate	20 (12.3)
high school graduation	75 (46.3)
no school graduation	3 (1.9)
missing	5
**Secondary education**	
apprenticeship	88 (53.3)
no further education after school graduation	7 (4.2)
university	60 (36.4)
other	10 (6.1)
missing	2
**Current job situation**	
employed	93 (57.4)
unemployed	59 (36.4)
in education	10 (6.2)
missing	5
**Retirement**	
no application	113 (76.9)
on retirement pension	13 (8.8)
disability pension	18 (12.2)
missing	23
**Currently on sick-leave**	
yes	55 (34.)
**Duration sick leave at presentation in weeks**	
mean (SD)	55.1 (71.6)
median (range)	32.5 (0–374)

The average number of symptoms reported by patients was *M* = 17.8 (*SD* = 10.5; range 2–46) with mean age at symptom onset of *M* = 34.9 years (*SD* = 17.7; range 0–70). The mean duration of the symptoms was *M* = 9.1 years (*SD* = 9.8; range 0–45 years). Comorbidities were reported by 142 patients (85.0%), of which 118 (83.1%) reported only somatic comorbidities and 24 (16.9%) both somatic and mental disorders, while none reported only mental comorbidities. Table 2 shows the most frequently reported comorbidities and the main symptoms causing discomfort.

**Table 2:** Comorbidities and most frequent symptoms causing main discomfort

Most common comorbidities reported by patients (*n* = 142)	n (%)	10 symptoms most frequently mentioned as main discomfort^1^ (*n* = 130)	n (%)
Allergies/ intolerances	71 (50.4)	Fatigue	37 (28.5)
Thyroid disease	48 (34.0)	Loss of productivity	28 (21.5)
Diseases of the skeletal system	47 (33.3)	Pain arms, hands, legs, feet	24 (18.5)
Respiratory disease	41 (29.1)	Muscular pain	20 (15.4)
Diseases of the digestive system	36 (25.5)	Joint pain	14 (10.8)
Eye disease	33 (23.4)	Irritation of the skin	14 (10.8)
Disease of the circulatory system	29 (20.6)	Muscle weakness	13 (10.0)
Renal and urinary tract disease	25 (17.7)	Stomachache	12 (9.2)
Neurological disease	25 (17.7)	Headache	12 (9.2)
Psychological disorder	24 (17.0)	Increased need for sleep	12 (9.2)
Heart disease	21 (14.9)	Dyspnea	11 (8.5)
Blood disease	20 (14.2)		
Metabolic disease	18 (12.8)		
Diseases of the muscular system	13 (9.2)		
Liver disease	8 (5.7)		

^1^ Patients were asked to name 3 symptoms mainly causing discomfort

Patients contacted between 1 and 25 different specialties before presenting at the MZCSE, with a mean of *M* = 10.3 (*SD* = 5.2). The majority of the patients (62.4%, *n* = 104) had been in inpatient care because of their symptoms, with 25 patients (15.9%) having spent 80 days or more in hospital. A total of 68 patients (43.0%) received at least one psychotherapeutic treatment in the past.

### Psychological distress

A total of *n* = 66 (40.7%; 95% CI:33.2–48.3) screened positive for any depressive disorder (PHQ-8 ≥ 10) and one quarter of the patients *n* = 44 (27.5%; 95% CI:20.6–34.4) screened positive for an anxiety disorder (GAD-7 ≥ 10). Further, a high share of *n* = 67 (45%; 95% CI:37.0–53.0) screened positive for SSD (PHQ-15 ≥ 9 & SSD-12 ≥ 23). Table 3 shows the psychopathological characteristics of our sample in combination with reference values from the general population and patients with the (self-reported) diagnosis of a rare disease.

**Table 3:** Psychological distress of adult patients presenting at a center for rare and undiagnosed diseases and comparative data

Psychological distress	Study sample	Patients diagnosed with a rare disease	General population
**Depression (PHQ-8)**			
mean (SD)	8.9 (5.2)		4.1 (3.9)^c^
range	0–24		
positive screening for a depressive disorder (PHQ-8 ≥ 10)	66 (40.7%)	42%^a^; 30%^b^	5.6%^d^
no significant depressive symptoms (0–4)	33 (20.4%)	34%^b^	76.4%^d^
mild (5–9)	63 (38.9%)	36%^b^	18.1%^d^
moderate (10–14)	45 (27.8%)	14%^b^	4.3%^d^
moderately severe (15–19)	15 (9.3%)	9%^b^	1.3%^d^
severe (20–24)	6 (3.7%)	7%^b^	
missing	5		
**Anxiety (GAD-7)**			
mean (SD)	6.4 (5.5)		3.36 (3.4)^c^
range	0–20		
positive screening for anxiety disorder (GAD-7 ≥ 10)	44 (27.5%)	23%^a^; 26%^b^	16.6%^e^
minimal (0–4)	77 (48.1%)	30%^b^	55.1%^e^
mild (5–9)	39 (24.4%)	44%^b^	28.1%^e^
moderate (10–14)	27 (16.9%)	14%^b^	9.6%^e^
severe (15–21)	17 (10.6%)	12%^b^	7.0%^e^
missing	7		
**Somatic symptom severity (PHQ-15)**			
mean (SD)	12.1 (5.8)		5.5 (3.9)^f^
range	0–26		
minimal (1–4)	13 (8.5%)		46.8%^f^
low (5–9)	41 (26.8%)		38.3%^f^
medium (10–14)	45 (29.4%)		11.8%^f^
high (15–30); (PHQ-15 ≥ 15)	53 (34.6%)		3.1%^f^
missing	14		
**Patients’ perceptions of their symptom-related thoughts, feelings, and behaviors (SSD-12)**			
mean (SD)	24.5 (10.8)		7.9 (9.3)^g^
range	0–47		0-48^ g^
high psychological burden (above 90% quantile cut-off of respective age-group)	93 (60%)		
missing	10		
**Positive screening for Somatic Symptom Disorder (PHQ-15 ≥ 9 & SSD-12 ≥ 23)**	67 (45%)		14,1%^h^
missing	18		

*Notes.* Source, sample size, instrument; data from Germany if not further specified: ^a^*n*=300, PHQ-9,GAD-7 [6]; ^b^*n*=86, PHQ-9, GAD-7, Spain [49]; ^c^*n*=113,928, PHQ-9, GAD-7 [50]; ^d^*n*=5018, PHQ-9 [34]; ^e^*n*=15,704, PHQ-2, GAD-7 [47]; ^f^*n*=9250, PHQ-15 [51];^g^*n*=2306, SSD-12 [37]; ^h^*n*=2531, SSS-8, WI-7 [52]

### Overlap between a positive screening for depression, anxiety and somatic symptom disorder

**Fig. 2:**
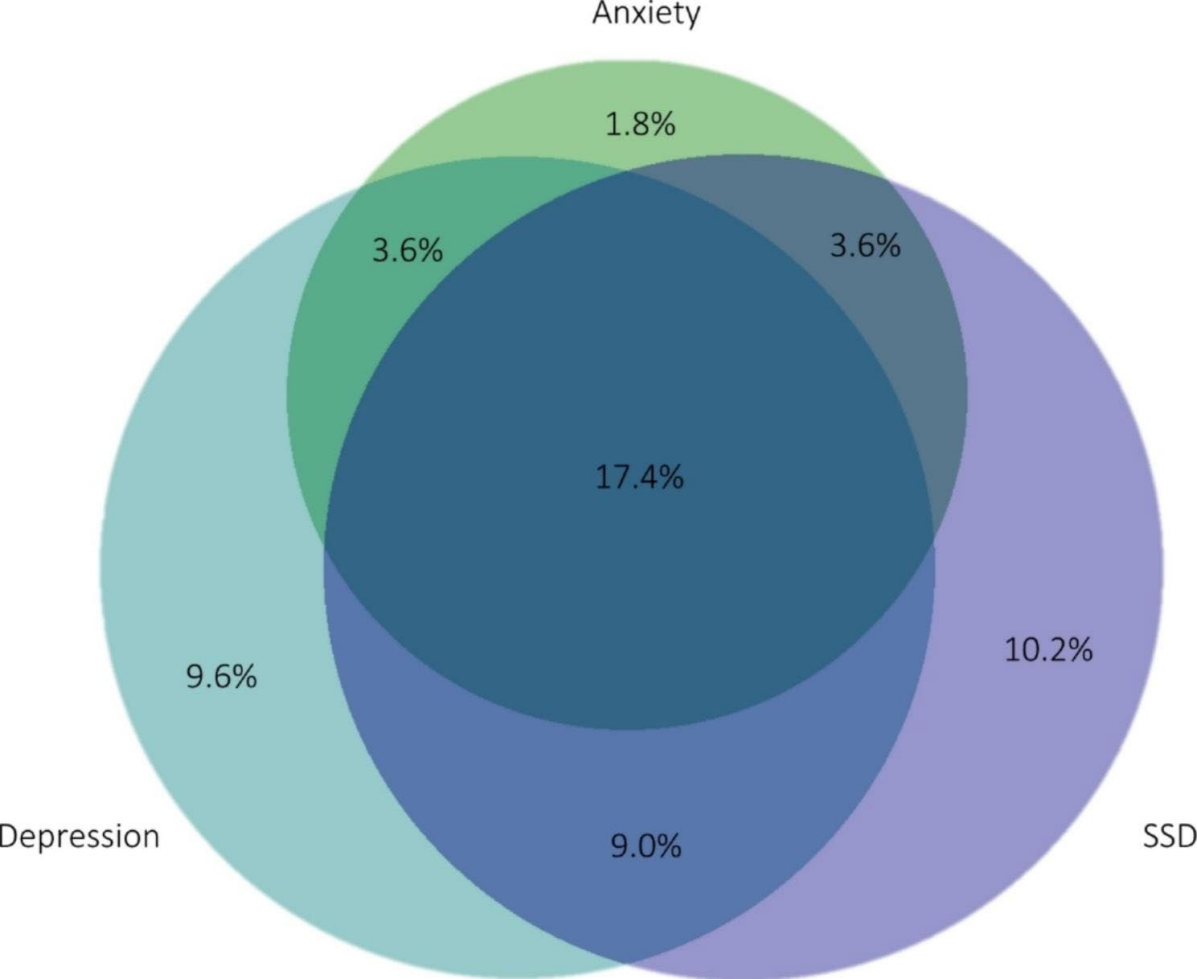
Overlap between a positive screening for a depressive disorder, an anxiety disorder, and SSD in an area-proportional Venn-Diagramm. (**Legend**: *N* = 167; Depression: PHQ8 ≥ 10, Anxiety: GAD7 ≥ 10, SSD: PHQ15 ≥ 9 & SSD12 ≥ 23)

*N* = 92 patients (55.1%; 95% CI:47.6–62.6) screened positive for at least one of these disorders. Of these, most patients screened positive for more than one diagnosis and *n* = 29 (17.4%; 95% CI:11.6–23.1) patients reached cut-off levels in all three screening instruments. *N* = 6 (3.6%; 95% CI:0.8–6.4) patients screened positive for both a depressive and anxiety disorder. The same number of patients screened positive for both an anxiety disorder and SSD and *n* = 15 (9.0%; 95% CI:4.7–13.3) screened positive for both a depressive disorder and SSD. *N* = 16 (9.6%; 95% CI:5.1–14.0) patients only screened positive for a depressive disorder. The same applies to *n* = 3 (1.8%; 95% CI:0.0–3.8) patients regarding anxiety disorders and *n* = 17 (10.2%; 95% CI:5.6–14.8) patients regarding SSD. Figure 2 shows the share of patients with the respective screening diagnoses and their overlap.

### Aspects associated with a positive screening for depression, anxiety, and somatic symptom disorder

Screening positive for a depressive disorder was associated with a higher number of reported symptoms (*U* = 4326.5, *p* < .001), a higher number of consulted specialties (*U* = 4216.5, *p* < .001), past psychotherapy (*p* < .001), reporting both somatic and psychological comorbidities (*p* = .001), not being employed (χ^2^ = 6.3, *p* = .043), and the number of days in stationary care (χ^2^ = 12.4, *p* = .030). Screening positive for an anxiety disorder was associated with the number of reported symptoms (*U* = 3186.0, *p* = .004), the number of consulted specialties (U = 3114.0, *p* = .031), past psychotherapy (*p* = .002), and secondary education (χ^2^ = 8.9, *p* = .012). Positive screening of SSD was associated with the number of reported symptoms (*U* = 1416.50, *p* < .001), the number of specialties visited (*U* = 1947.5, *p* = .002), past psychotherapy (*p* < .001), secondary education (χ^2^ = 10.4, *p* = .006), unemployment (χ^2^ = 7.1, *p* = .028), and sick leave (*p* = .001). Gender, age at presentation, age at symptom onset, duration of the symptoms, whether patients reported comorbidities, retirement status, and the number of stationary stays were not associated with increased psychopathology in any of the three dimensions. An overview of all calculated associations can be found in Tables 4, 5 and 6 of the supplementary material.

## Discussion

To our knowledge, this is the first study systematically assessing psychological distress using the PHQ-8, GAD-7, PHQ-15 and SSD-12 in adult patients consulting a center for rare diseases in Germany. The majority of the patients had increased psychopathology levels, with 40% screening positive for a depressive disorder, about a quarter screening positive for an anxiety disorder, and 45% screening positive for SSD. The findings support our assumption that patients with an unclear diagnosis consulting a center for rare diseases are a vulnerable patient population with regard to psychological distress.

The sociodemographic and clinical characteristics are largely consistent with other studies characterizing patients at rare diseases centers in Germany [2, 4]. In accordance with Mueller and colleagues [2], patients presented with heterogeneous complaints and often unspecific symptoms such as fatigue, loss of productivity, and pain. Levels of self-reported comorbidities were very high, with the majority being somatic conditions. Roughly one third of the patients were on sick leave at the time of presentation, about 36% were unemployed and 12% were on disability pension. For comparison, in the German general population there were 6.1% on sick leave in February 2022 [41], and 5.4% are currently unemployed [42]. In 2020, of all federal pension insured civilists 2.4% were on disability pension [43], indicating substantially restricted functionality of our sample.

The percentages of patients screening positive for depressive and anxiety disorders were higher compared to population-based samples and similar to those found in patients with a diagnosis of a rare disease (see Table 3). This stresses that patients with undiagnosed diseases experience similar psychological distress as patients with severe and disabling rare diseases. A total of 45% of the patients screened positive for a SSD. In a scoping review synthesizing empirical evidence on SSD, the mean prevalence of SSD was about 13% in the general population, about 25% in patients with various somatic conditions, and similarly high in patients with medically unexplained symptoms [20]. In line with Löwe and colleagues [44], the majority of the patients of our sample showed overlap between the screening diagnoses of depressive and anxiety disorders and SSD. Comorbid anxiety, depression, and somatization can drive functional impairment exceedingly [44]. It should be noted that screening positive for a depressive or anxiety disorder or SSD does not provide any information about whether or not patients have a rare somatic disease.

This is the first study investigating SSD in the context of rare diseases. The earlier diagnostic concepts of somatoform or psychosomatic disorders in DSM-IV and ICD-10 have been criticized for fostering a body-mind dualism as the exclusion of a medical explanation for the symptoms was a necessary precondition for diagnosis [20]. Body-mind dualism carries the risk of understating mental distress once a somatic diagnosis has been identified, or conversely, of stopping the search for a somatic diagnosis once a patient is labelled as mentally ill. SSD explicitly demands an evaluation of psychological distress regardless of any (potentially) underlying somatic disorder, and therefore enables taking into account both somatic and psychological factors. Besides a more precise diagnosis, this can help to reduce stigmatization of patients.

We exploratorily investigated aspects that are associated with psychological distress and found associations with a higher number of different symptoms, the number of specialties consulted before presenting at the MZCSE as well as past psychotherapy. Moreover, we found associations between psychopathology and socioeconomical and work-related aspects including education and employment. The relationship between lower educational and socioecomic status and mental health can be considered a scientific consensus [45]. The number of specialties that patients had consulted before presenting at the MZCSE may be an indicator for the length of the diagnostic journey that patients have already undertaken and the result could reflect the psychological burden this path can cause. This is in line with qualitative studies on difficulties that patients with rare diseases experience [10, 11].

This study has some limitations. Firstly, due to the cross-sectional design, no conclusions about causal relationships between any of the variables can be made. Secondly, all variables were assessed via self-report. Aspects that require reminding past events, such as the number of specialties, may be subject to a recall bias. Thirdly, patients knew that their responses were considered in the diagnostic process, which might have influenced their responses. In addition, the reported comorbidities are not confirmed by a physician and may include self-diagnoses. Moreover, psychopathology was assessed with screening instruments. The self-report questionnaires we used are well validated and allow a reliable assessment of psychopathological symptom severity. However, it can be criticized that screening instruments could lead to an overestimation of prevalence rates and do not replace a clinical diagnosis. Moreover, among the very heterogeneous population of patients with rare diseases, it is possible that those with more severe symptoms are more likely to present at a center for rare diseases, resulting in a possible pre-selection. Furthermore, our results are a single-center oberservation. Generalizability to other centers for rare diseases is unclear. Lastly, data collection took place during the Covid-19 pandemic, which may have impacted our results. Many patients with rare diseases reported interruptions in their regular healthcare, which may have led to a further disadvantage of an already vulnerable group [46]. It is unclear whether there are patients who had limited access to primary care and therefore did not find their way to our center. Moreover, anxiety and depression rates increased due to the pandemic [47], which may also have affected the findings of our study. Where possible, we used comparison data collected during the same period, which helps to contextualize our findings. Still, patients may have been particularly vulnerable to psychological distress during the time we conducted this study.

## Conclusions

Our results support the assumption that patients presenting at centers for rare diseases are likely to experience psychological distress. Regardless of whether the diagnostic procedure at the center results in diagnosing a rare disease, early detection and treatment of mental diseases can be crucial for patients’ well-being and quality of life. Systematically applying standardized screening instruments such as the PHQ-8 [24], GAD-7 [28], PHQ-15 [31, 32] and SSD-12 [35] in routine diagnostic procedures could help to identify patients at risk earlier and reduce psychopathology, as for instance demonstrated for depression severity in cardiac patients [48]. Relying on the new diagnostic concept of SSD when screening for psychopathology can help to ensure a precise evaluation of patients’ burden beyond body-mind dualism. Once detected, interdisciplinary cooperation is crucial to initate adequate care for patients with increased psychopathology levels. Integrating experts in psychosomatic medicine or consultation-liaison psychiatry into routine prodecures at rare disease centers can help to ensure patients receive the support they need.

## Electronic supplementary material

Below is the link to the electronic supplementary material.

Supplementary Material 1

## Data Availability

The datasets used and analyzed during the current study are available from the corresponding author on reasonable request.

## List of abbreviations

GAD-7: Generalized Anxiety Disorder 7-item scale
MZCSE: Martin Zeitz Center for Rare Diseases
PHQ: Patient Health Questionnaire
SSD: Somatic symptom disorder
SSD-12: Somatic Symptom Disorder-B Criteria Scale
STROBE: STrengthening the Reporting of OBservational studies in Epidemiology

## References

[1] Khosla N, Valdez R (2018). A compilation of national plans, policies and government actions for rare diseases in 23 countries. Intractable Rare Dis Res.

[2] Mueller T, Jerrentrup A, Bauer MJ, Fritsch HW, Schaefer JR (2016). Characteristics of patients contacting a center for undiagnosed and rare diseases. Orphanet J Rare Dis.

[3] Waserstein G, Partin C, Cohen D, Schettler P, Kinkead B, Rapaport MH (2019). The prevalence and impact of psychiatric symptoms in an undiagnosed diseases clinical program. PLoS ONE.

[4] Rillig F, Grüters A, Bäumer T, Hoffmann GF, Choukair D, Berner R (2022). The interdisciplinary diagnosis of rare diseases - results of the Translate-NAMSE project. Dtsch Arztebl Int.

[5] Uhlenbusch N, Swaydan J, Höller A, Löwe B, Depping MK (2021). Affective and anxiety disorders in patients with different rare chronic diseases: a systematic review and meta-analysis. Psychol Med.

[6] Uhlenbusch N, Löwe B, Härter M, Schramm C, Weiler-Normann C, Depping MK (2019). Depression and anxiety in patients with different rare chronic diseases: a cross-sectional study. PLoS ONE.

[7] EURORDIS. What is a rare disease? 2009 [updated July 21., 2020. Available from: https://www.eurordis.org/content/what-rare-disease.

[8] Schieppati A, Henter JI, Daina E, Aperia A (2008). Why rare diseases are an important medical and social issue. Lancet.

[9] EURORDIS. EurordisCare 2: Survey of the delay in diagnosis for 8 rare diseases in Europe. European Organisation for Rare Diseases. ; 2017 Apr 7, 2017.

[10] Uhlenbusch N, Löwe B, Depping MK (2019). Perceived burden in dealing with different rare diseases: a qualitative focus group study. BMJ Open.

[11] von der Lippe C, Diesen PS, Feragen KB (2017). Living with a rare disorder: a systematic review of the qualitative literature. Mol Genet Genomic Med.

[12] Katon W, Lin EH, Kroenke K (2007). The association of depression and anxiety with medical symptom burden in patients with chronic medical illness. Gen Hosp Psychiatry.

[13] Blakemore A, Dickens C, Guthrie E, Bower P, Kontopantelis E, Afzal C (2014). Depression and anxiety predict health-related quality of life in chronic obstructive pulmonary disease: systematic review and meta-analysis. Int J Chron Obstruct Pulmon Dis.

[14] Schram MT, Baan CA, Pouwer F (2009). Depression and quality of life in patients with diabetes: a systematic review from the european depression in diabetes (EDID) research consortium. Curr Diabetes Rev.

[15] Escobar JI, Cook B, Chen CN, Gara MA, Alegría M, Interian A (2010). Whether medically unexplained or not, three or more concurrent somatic symptoms predict psychopathology and service use in community populations. J Psychosom Res.

[16] Löwe B, Andresen V, Van den Bergh O, Huber TB, von dem Knesebeck O, Lohse AW (2022). Persistent SOMAtic symptoms ACROSS diseases - from risk factors to modification: scientific framework and overarching protocol of the interdisciplinary SOMACROSS research unit (RU 5211). BMJ Open.

[17] Löwe B, Spitzer RL, Williams JB, Mussell M, Schellberg D, Kroenke K (2008). Depression, anxiety and somatization in primary care: syndrome overlap and functional impairment. Gen Hosp Psychiatry.

[18] Klaus K, Rief W, Brähler E, Martin A, Glaesmer H, Mewes R (2013). The distinction between “medically unexplained” and “medically explained” in the context of somatoform disorders. Int J Behav Med.

[19] Joustra ML, Janssens KA, Bültmann U, Rosmalen JG (2015). Functional limitations in functional somatic syndromes and well-defined medical diseases. Results from the general population cohort LifeLines. J Psychosom Res.

[20] Löwe B, Levenson J, Depping M, Hüsing P, Kohlmann S, Lehmann M (2021). Somatic symptom disorder: a scoping review on the empirical evidence of a new diagnosis. Psychol Med.

[21] Llubes-Arrià L, Sanromà-Ortíz M, Torné-Ruiz A, Carillo-Álvarez E, García-Expósito J, Roca J (2022). Emotional experience of the diagnostic process of a rare disease and the perception of support systems: a scoping review. J Clin Nurs.

[22] von Elm E, Altman DG, Egger M, Pocock SJ, Gøtzsche PC, Vandenbroucke JP (2014). The strengthening the reporting of Observational Studies in Epidemiology (STROBE) Statement: guidelines for reporting observational studies. Int J Surg.

[23] Löwe B, Spitzer RL, Gräfe K, Kroenke K, Quenter A, Zipfel S (2004). Comparative validity of three screening questionnaires for DSM-IV depressive disorders and physicians’ diagnoses. J Affect Disord.

[24] Kroenke K, Strine TW, Spitzer RL, Williams JB, Berry JT, Mokdad AH (2009). The PHQ-8 as a measure of current depression in the general population. J Affect Disord.

[25] Pressler SJ, Subramanian U, Perkins SM, Gradus-Pizlo I, Kareken D, Kim J (2011). Measuring depressive symptoms in heart failure: validity and reliability of the patient health questionnaire-8. American journal of critical care: an official publication. Am Association Critical-Care Nurses.

[26] Shin C, Lee SH, Han KM, Yoon HK, Han C (2019). Comparison of the usefulness of the PHQ-8 and PHQ-9 for screening for major depressive disorder: analysis of Psychiatric Outpatient Data. Psychiatry Investig.

[27] McGuire LC, Strine TW, Allen RS, Anderson LA, Mokdad AH (2009). The Patient Health Questionnaire 8: current depressive symptoms among U.S. older adults, 2006 behavioral risk factor Surveillance System. Am J geriatric psychiatry: official J Am Association Geriatric Psychiatry.

[28] Spitzer RL, Kroenke K, Williams JB, Löwe B (2006). A brief measure for assessing generalized anxiety disorder: the GAD-7. Arch Intern Med.

[29] Löwe B, Decker O, Müller S, Brähler E, Schellberg D, Herzog W (2008). Validation and standardization of the generalized anxiety disorder screener (GAD-7) in the general population. Med Care.

[30] Kroenke K, Spitzer RL, Williams JB, Monahan PO, Löwe B (2007). Anxiety disorders in primary care: prevalence, impairment, comorbidity, and detection. Ann Intern Med.

[31] Kroenke K, Spitzer RL, Williams JB (2002). The PHQ-15: validity of a new measure for evaluating the severity of somatic symptoms. Psychosom Med.

[32] Gräfe K, Zipfel S, Herzog W, Löwe B (2004). Screening psychischer Störungen mit dem “Gesundheitsfragebogen für Patienten (PHQ-D)“ [Screening for psychiatric disorders with the Patient Health Questionnaire (PHQ). Results from the german validation study]. Diagnostica.

[33] Kroenke K, Spitzer RL, Williams JB, Löwe B (2010). The Patient Health Questionnaire somatic, anxiety, and depressive Symptom Scales: a systematic review. Gen Hosp Psychiatry.

[34] Kocalevent RD, Hinz A, Brähler E (2013). Standardization of the depression screener patient health questionnaire (PHQ-9) in the general population. Gen Hosp Psychiatry.

[35] Toussaint A, Murray AM, Voigt K, Herzog A, Gierk B, Kroenke K (2016). Development and validation of the somatic Symptom Disorder-B criteria scale (SSD-12). Psychosom Med.

[36] Toussaint A, Riedl B, Kehrer S, Schneider A, Löwe B, Linde K (2018). Validity of the somatic Symptom Disorder-B criteria scale (SSD-12) in primary care. Fam Pract.

[37] Toussaint A, Löwe B, Brähler E, Jordan P (2017). The somatic Symptom disorder - B criteria scale (SSD-12): factorial structure, validity and population-based norms. J Psychosom Res.

[38] Toussaint A, Hüsing P, Kohlmann S, Löwe B (2020). Detecting DSM-5 somatic symptom disorder: criterion validity of the Patient Health Questionnaire-15 (PHQ-15) and the somatic Symptom Scale-8 (SSS-8) in combination with the somatic Symptom disorder - B criteria scale (SSD-12). Psychol Med.

[39] IBM Corp (2020). IBM SPSS Statistics for Windows. 27.0 ed.

[40] Hulsen T, de Vlieg J, Alkema W (2008). BioVenn - a web application for the comparison and visualization of biological lists using area-proportional Venn diagrams. BMC Genomics.

[41] Statista. Monatlicher Krankenstand der Mitglieder in der GKV nach Geschlecht in den Monaten Februar 2021 bis Februar 2022.BMG; 2022 Mar 31, 2022.

[42] Statista. Arbeitslosenquote in Deutschland im Jahresdurchschnitt von 2005 bis 2022. Bundesagentur für Arbeit; 2022. Mar 31, 2022.

[43] Deutsche Rentenversicherung Bund. Rentenbestand in Deutschland von 1990 bis 2020 nach Rentengrund. 2022 Mar 2022.

[44] Löwe B, Spitzer RL, Williams JB, Mussel M, Schellberg D, Kroenke K. Depression, anxiety and somatization in primary care: syndrome overlap and functional impairment. Gen Hosp Psychiatry. 2008;30(3):191-9 10.1016/j.genhosppsych.2008.01.001 18433651

[45] Macintyre A, Ferris D, Gonçalves B, Quinn N (2018). What has economics got to do with it? The impact of socioeconomic factors on mental health and the case for collective action. Palgrave Commun.

[46] Chowdhury SF, Sium SMA, Anwar S. Research and Management of Rare Diseases in the COVID-19 Pandemic Era: Challenges and Countermeasures.Frontiers in Public Health. 2021;9. 10.3389/fpubh.2021.640282 PMC8082070 33937170

[47] Bäuerle A, Teufel M, Musche V, Weismüller B, Kohler H, Hetkamp M (2020). Increased generalized anxiety, depression and distress during the COVID-19 pandemic: a cross-sectional study in Germany. J Public Health (Oxf).

[48] Löwe B, Blankenberg S, Wegscheider K, König HH, Walter D, Murray AM (2017). Depression screening with patient-targeted feedback in cardiology: DEPSCREEN-INFO randomised clinical trial. Br J Psychiatry.

[49] Sánchez-García JC, Cortés-Martín J, Rodríguez-Blanque R, Marín-Jiménez AE, Montiel-Troya M, Díaz-Rodríguez L (2021). Depression and anxiety in patients with Rare Diseases during the COVID-19 pandemic. Int J Environ Res Public Health.

[50] Peters A, Rospleszcz S, Greiser KH, Dallavalle M, Berger K (2020). The impact of the COVID-19 pandemic on self-reported health – early evidence from the German National Cohort. Dtsch Arztebl Int.

[51] Hinz A, Ernst J, Glaesmer H, Brähler E, Rauscher FG, Petrowski K (2017). Frequency of somatic symptoms in the general population: normative values for the Patient Health Questionnaire-15 (PHQ-15). J Psychosom Res.

[52] Hauser W, Hausteiner-Wiehle C, Henningsen P, Brahler E, Schmalbach B, Wolfe F (2020). Prevalence and overlap of somatic symptom disorder, bodily distress syndrome and fibromyalgia syndrome in the german general population: a cross sectional study. J Psychosom Res.

