# Supplementary material for: Psychological distress of adult patients consulting a center for rare and undiagnosed diseases: a cross-sectional study
Source: Orphanet J Rare Dis. 2023 Apr 14;18:82. doi: 10.1186/s13023-023-02669-7 (PMC10103043; doi:10.1186/s13023-023-02669-7)
Supplement: Supplementary file 1 — Supplementary Material 1 [file 13023_2023_2669_MOESM1_ESM.docx]

**Table 4.** Association between a positive screening for depression (PHQ-8) and patient characteristics.

| **Variable** | **PHQ-8** | ***n*** | ***Md*** | ***M*** | | ***SD*** | | ***U*** | ***Z*** | ***p*** |
| --- | --- | --- | --- | --- | --- | --- | --- | --- | --- | --- |
| Age at presentation | <10 | 96 | 48.00 | 46.55 | | 13.44 | | 2594.50 | -1.96 | .051 |
|  | ≥10 | 66 | 41.00 | 42.33 | | 14.77 | |  |  |  |
| Duration of symptoms | <10 | 89 | 6.00 | 8.48 | | 9.64 | | 2702.00 | -0.22 | .828 |
|  | ≥10 | 62 | 4.00 | 8.49 | | 9.61 | |  |  |  |
| Number of symptoms | <10 | 94 | 13.00 | 14.77 | | 9.29 | | 4326.50 | 4.46 | **.000** |
|  | ≥10 | 65 | 23.00 | 22.28 | | 10.53 | |  |  |  |
| Age at disease onset | <10 | 89 | 40.00 | 37.80 | | 15.42 | | 2397.50 | -1.37 | .171 |
|  | ≥10 | 62 | 30.00 | 33.95 | | 18.74 | |  |  |  |
| Number of specialties consulted before | <10 | 96 | 8.00 | 9.39 | | 4.72 | | 4216.50 | 3.58 | **.000** |
|  | ≥10 | 66 | 11.00 | 11.84 | | 5.11 | |  |  |  |
| **Variable** |  | | | ***n*** | | | ***N*** | ***χ^2^*** | ***df*** | ***p*** |
|  |  |  |  | <10 | ≥10 | |  |  |  |  |
| Gender | Male | | | 30 | 26 | | 161 |  |  | .312 |
|  | Female | | | 66 | 39 | |  |  |  |  |
| Psychotherapy | No | | | 67 | 21 | | 156 |  |  | **.000** |
|  | Yes | | | 26 | 42 | |  |  |  |  |
| Primary education | Graduation after 9th grade | | | 12 | 10 | | 156 | 3.34 | 3 | .342 |
|  | Graduation after 10th grade | | | 20 | 20 | |  |  |  |  |
|  | Technical baccalaureate | | | 13 | 7 | |  |  |  |  |
|  | High school graduation | | | 49 | 25 | |  |  |  |  |
| Secondary education | University | | | 40 | 19 | | 161 | 4.38 | 2 | .112 |
|  | Apprenticeship | | | 49 | 38 | |  |  |  |  |
|  | No secondary education/other | | | 6 | 9 | |  |  |  |  |
| Current employment status | Employed | | | 61 | 29 | | 158 | 6.31 | 2 | **.043** |
|  | Unemployed | | | 29 | 29 | |  |  |  |  |
|  | In education | | | 4 | 6 | |  |  |  |  |
| Current sick leave | Yes | | | 28 | 27 | | 158 |  |  | .127 |
|  | No | | | 66 | 37 | |  |  |  |  |
| Pension status | No pension application filed | | | 69 | 39 | | 139 | 2.46 | 2 | .293 |
|  | Retirement pension | | | 8 | 5 | |  |  |  |  |
|  | Disability pension | | | 8 | 10 | |  |  |  |  |
| Comorbidities | Yes | | | 82 | 56 | | 162 |  |  | >.999 |
|  | No | | | 14 | 10 | |  |  |  |  |
| Comorbidities | Psycholog. & somatic | | | 7 | 17 | | 138 |  |  | **.001** |
|  | Only somatic | | | 75 | 39 | |  |  |  |  |
| Frequency of inpatient treatment | None | | | 39 | 19 | | 159 | 4.23 | 4 | .375 |
|  | 1 | | | 14 | 8 | |  |  |  |  |
|  | 2 - 3 | | | 16 | 14 | |  |  |  |  |
|  | 4 - 8 | | | 14 | 16 | |  |  |  |  |
|  | More than 8 | | | 12 | 7 | |  |  |  |  |
| Total days in inpatient treatment | None | | | 35 | 19 | | 154 | 12.35 | 5 | **.030** |
|  | 1 - 10 | | | 26 | 8 | |  |  |  |  |
|  | 11 - 20 | | | 4 | 9 | |  |  |  |  |
|  | 21 - 30 | | | 10 | 7 | |  |  |  |  |
|  | 31 - 80 | | | 7 | 5 | |  |  |  |  |
|  | More than 80 | | | 10 | 14 | |  |  |  |  |

**Table 5.** Association between a positive screening for anxiety (GAD-7) and patient characteristics.

| **Variable** | **GAD-7** | ***n*** | ***Md*** | ***M*** | | ***SD*** | | ***U*** | ***Z*** | ***p*** |
| --- | --- | --- | --- | --- | --- | --- | --- | --- | --- | --- |
| Age at presentation | <10 | 116 | 45.00 | 44.71 | | 14.57 | | 2494.00 | -0.22 | .825 |
|  | ≥10 | 44 | 43.00 | 43.70 | | 13.33 | |  |  |  |
| Duration of symptoms | <10 | 109 | 5.00 | 8.30 | | 8.93 | | 2292.50 | 0.25 | .806 |
|  | ≥10 | 41 | 5.00 | 10.20 | | 11.46 | |  |  |  |
| Number of symptoms | <10 | 114 | 14.00 | 16.06 | | 9.86 | | 3186.00 | 2.90 | **.004** |
|  | ≥10 | 43 | 22.00 | 22.50 | | 10.72 | |  |  |  |
| Age at disease onset | <10 | 109 | 36.00 | 36.38 | | 16.80 | | 1990.50 | -1.03 | .303 |
|  | ≥10 | 41 | 33.00 | 33.15 | | 17.73 | |  |  |  |
| Number of specialties consulted before | <10 | 116 | 9.00 | 9.81 | | 4.71 | | 3114.00 | 2.15 | **.031** |
|  | ≥10 | 44 | 11.00 | 12.13 | | 5.50 | |  |  |  |
| **Variable** |  | | | ***n*** | | | ***N*** | ***χ^2^*** | ***df*** | ***p*** |
|  |  |  |  | <10 | ≥10 | |  |  |  |  |
| Gender | Male | | | 41 | 14 | | 159 |  |  | .712 |
|  | Female | | | 74 | 30 | |  |  |  |  |
| Psychotherapy | No | | | 72 | 15 | | 153 |  |  | **.002** |
|  | Yes | | | 39 | 27 | |  |  |  |  |
| Primary education | Graduation after 9th grade | | | 12 | 9 | | 153 | 4.30 | 3 | .231 |
|  | Graduation after 10th grade | | | 28 | 13 | |  |  |  |  |
|  | Technical baccalaureate | | | 16 | 4 | |  |  |  |  |
|  | High school graduation | | | 55 | 16 | |  |  |  |  |
| Secondary education | University | | | 46 | 13 | | 159 | 8.90 | 2 | **.012** |
|  | Apprenticeship | | | 63 | 22 | |  |  |  |  |
|  | No secondary education/other | | | 6 | 9 | |  |  |  |  |
| Current employment status | Employed | | | 69 | 22 | | 156 | 1.53 | 2 | .464 |
|  | Unemployed | | | 37 | 18 | |  |  |  |  |
|  | In education | | | 8 | 2 | |  |  |  |  |
| Current sick leave | Yes | | | 36 | 19 | | 155 |  |  | .134 |
|  | No | | | 77 | 23 | |  |  |  |  |
| Pension status | No pension application filed | | | 80 | 29 | | 139 | 5.88 | 2 | .053 |
|  | Retirement pension | | | 12 | 1 | |  |  |  |  |
|  | Disability pension | | | 9 | 8 | |  |  |  |  |
| Cormorbidities | Yes | | | 98 | 39 | | 160 |  |  | .348 |
|  | No | | | 18 | 5 | |  |  |  |  |
| Comorbidities | Psycholog. & somatic | | | 14 | 10 | | 137 |  |  | .137 |
|  | Only somatic | | | 84 | 29 | |  |  |  |  |
| Frequency of inpatient treatment | None | | | 41 | 16 | | 156 | 6.36 | 4 | .174 |
|  | 1 | | | 19 | 4 | |  |  |  |  |
|  | 2 - 3 | | | 24 | 4 | |  |  |  |  |
|  | 4 - 8 | | | 18 | 12 | |  |  |  |  |
|  | More than 8 | | | 12 | 6 | |  |  |  |  |
| Total days in inpatient treatment | None | | | 37 | 16 | | 151 | 6.74 | 5 | .241 |
|  | 1 - 10 | | | 29 | 4 | |  |  |  |  |
|  | 11 - 20 | | | 9 | 3 | |  |  |  |  |
|  | 21 - 30 | | | 12 | 5 | |  |  |  |  |
|  | 31 - 80 | | | 8 | 4 | |  |  |  |  |
|  | More than 80 | | | 14 | 10 | |  |  |  |  |

**Table 6.** Association between a positive screening for a somatic symptom disorder (SSD) and patient characteristics.

| **Variable** | **Indication of SSD^1^** | ***n*** | ***Md*** | ***M*** | | ***SD*** | | ***U*** | ***Z*** | ***p*** |
| --- | --- | --- | --- | --- | --- | --- | --- | --- | --- | --- |
| Age at presentation | no | 82 | 45.00 | 45.00 | | 13.96 | | 2639.50 | -0.41 | .682 |
|  | yes | 67 | 43.00 | 43.92 | | 14.15 | |  |  |  |
| Duration of symptoms | no | 75 | 6.00 | 9.59 | | 9.72 | | 1952.00 | -1.90 | .057 |
|  | yes | 64 | 3.00 | 6.87 | | 8.72 | |  |  |  |
| Number of symptoms | no | 81 | 13.00 | 14.49 | | 8.78 | | 3929.50 | 4.90 | **.000** |
|  | yes | 66 | 23.00 | 22.81 | | 10.32 | |  |  |  |
| Age at disease onset | no | 75 | 37.50 | 35.51 | | 17.07 | | 2418.50 | 0.08 | .938 |
|  | yes | 64 | 36.00 | 36.67 | | 16.53 | |  |  |  |
| Number of specialties consulted before | no | 82 | 9.00 | 9.55 | | 4.70 | | 3546.50 | 3.06 | **.002** |
|  | yes | 67 | 11.00 | 11.89 | | 5.07 | |  |  |  |
| **Variable** |  | | | ***n*** | | | ***N*** | ***χ^2^*** | ***df*** | ***p*** |
|  |  |  |  | **Indication of SSD** | | |  |  |  |  |
|  |  |  |  | **no** | **yes** | |  |  |  |  |
| Gender | Male | | | 28 | 19 | | 148 |  |  | .594 |
|  | Female | | | 54 | 47 | |  |  |  |  |
| Psychotherapy | No | | | 57 | 25 | | 145 |  |  | **.000** |
|  | Yes | | | 24 | 39 | |  |  |  |  |
| Primary education | Graduation after 9th grade | | | 7 | 12 | | 145 | 4.76 | 3 | .191 |
|  | Graduation after 10th grade | | | 24 | 16 | |  |  |  |  |
|  | Technical baccalaureate | | | 8 | 8 | |  |  |  |  |
|  | High school graduation | | | 43 | 26 | |  |  |  |  |
| Secondary education | University | | | 35 | 19 | | 149 | 10.35 | 2 | **.006** |
|  | Apprenticeship | | | 45 | 37 | |  |  |  |  |
|  | No secondary education/other | | | 2 | 11 | |  |  |  |  |
| Current employment status | Employed | | | 55 | 31 | | 146 | 7.12 | 2 | **.028** |
|  | Unemployed | | | 21 | 30 | |  |  |  |  |
|  | In education | | | 4 | 5 | |  |  |  |  |
| Current sick leave | Yes | | | 18 | 32 | | 146 |  |  | **.001** |
|  | No | | | 63 | 33 | |  |  |  |  |
| Pension status | No pension application filed | | | 60 | 42 | | 130 | 3.55 | 2 | .170 |
|  | Retirement pension | | | 7 | 4 | |  |  |  |  |
|  | Disability pension | | | 6 | 11 | |  |  |  |  |
| Comorbidities | Yes | | | 71 | 57 | | 149 |  |  | .817 |
|  | No | | | 11 | 10 | |  |  |  |  |
| Comorbidities | Psycholog. & somatic | | | 9 | 15 | | 128 |  |  | .068 |
|  | Only somatic | | | 62 | 42 | |  |  |  |  |
| Frequency of inpatient treatment | None | | | 32 | 20 | | 146 | 7.05 | 4 | .133 |
|  | 1 | | | 14 | 7 | |  |  |  |  |
|  | 2 - 3 | | | 16 | 12 | |  |  |  |  |
|  | 4 - 8 | | | 10 | 19 | |  |  |  |  |
|  | More than 8 | | | 9 | 7 | |  |  |  |  |
| Total days in inpatient treatment | None | | | 29 | 19 | | 142 | 4.94 | 5 | .423 |
|  | 1 - 10 | | | 20 | 10 | |  |  |  |  |
|  | 11 - 20 | | | 5 | 8 | |  |  |  |  |
|  | 21 - 30 | | | 8 | 9 | |  |  |  |  |
|  | 31 - 80 | | | 5 | 7 | |  |  |  |  |
|  | More than 80 | | | 12 | 10 | |  |  |  |  |

*Notes*. ^1^SSD indicated by PHQ15 ≥ 9 & SSD12 ≥ 23.
